# From Green Extraction to Gut Bioaccessibility: Synergistic Potential of *Ginkgo biloba*, *Astragalus membranaceus*, and *Salvia miltiorrhiza* Phytochemicals for Functional Food Applications

**DOI:** 10.1002/fsn3.72045

**Published:** 2026-06-29

**Authors:** Mohamed Ibrahim Younis, Yahia Ibrahim Sallam, Khaled Fahmy Mahmoud, Rawaa H. Tlay, M. Ali Aboudzadeh, Tarek Gamal Abedelmaksoud

**Affiliations:** ^1^ Food Science Department, Faculty of Agriculture Cairo University Giza Egypt; ^2^ Food Technology Department National Research Centre Giza Egypt; ^3^ Department of Food Science, College of Agricultural Engineering Damascus University Damascus Syria; ^4^ POLYMAT and Department of Applied Chemistry, Faculty of Chemistry University of the Basque Country UPV/EHU Donostia‐San Sebastián Spain

**Keywords:** anti‐inflammatory, antioxidant activity, bio‐accessibility, cytotoxicity, green extraction

## Abstract

Green extraction technologies offer sustainable alternatives to conventional solvent‐based methods for recovering phytochemicals from medicinal plants. In this study, different blends of *Ginkgo biloba* leaves, *Astragalus membranaceus* roots, and *Salvia miltiorrhiza* roots were screened, and the 1:1:2 blend showed the strongest TPC‐based apparent synergistic effect on total phenolic content (TPC). Probe ultrasound‐assisted extraction (probe‐UAE), bath ultrasound‐assisted extraction, and microwave‐assisted extraction were then optimized using response surface methodology. Probe‐UAE yielded the highest TPC, with an experimental value of 342.73 mg GAE/g under the optimized conditions. HPLC analysis of the prevailing synergistic extract (PSE) revealed rosmarinic acid (249.58 μg/mL), chlorogenic acid (135.59 μg/mL), and catechin (36.93 μg/mL) as the predominant identified phenolic compounds. The PSE showed strong antioxidant activity (DPPH IC_50_ = 23.04 μg/mL; ABTS IC_50_ = 30.71 μg/mL; FRAP/reducing power = 260.27 μg AAE/mg extract; TAC = 345.20 μg AAE/mg extract), anti‐inflammatory activity (protein denaturation IC_50_ = 18.21 μg/mL), and α‐amylase inhibition (IC_50_ = 40.58 μg/mL). The extract also exhibited antimicrobial activity, with the highest inhibition zone against *Staphylococcus aureus* (30 mm; MIC = 15.62 μg/mL), and moderate cytotoxic activity against PC3 and MCF‐7 cells (IC_50_ = 84.82 and 109.76 μg/mL, respectively). Simulated gastrointestinal digestion showed that total phenolics and flavonoids remained bioaccessible across digestive phases, although phenolics decreased progressively toward the intestinal phase. Overall, the optimized tri‐herbal extract demonstrated multifunctional in vitro bioactivity and may represent a promising candidate for functional food and nutraceutical applications. Further in vivo validation and formulation studies are warranted.

## Graphical abstract

This study optimized green extraction of a synergistic tri‐herbal blend of *Ginkgo biloba* , *Astragalus membranaceus*, and *Salvia miltiorrhiza* . The 1:1:2 blend showed the highest TPC synergy, with probe ultrasound giving maximal phenolic recovery. The extract, rich in rosmarinic acid, chlorogenic acid, and catechin, exhibited antioxidant, antimicrobial, anti‐inflammatory, cytotoxic, and bioaccessibility.

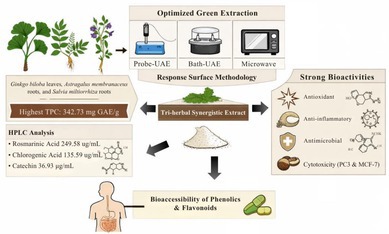

## 1 Introduction

Medicinal herbs have been used therapeutically for ages and are now being considered for food purposes. These plants promote health due to bioactive substances such as polyphenols, alkaloids, terpenoids, and flavonoids (Ali et al. 2025). Antioxidant, anti‐inflammatory, antibacterial, and immune‐boosting qualities make medicinal plants valuable for illness prevention and treatment (Dabas et al. 2023). In the food industry, functional foods, beverages, and dietary supplements use these ingredients to boost nutritional value and provide health advantages beyond basic nutrition (Mittal et al. 2024).

*Ginkgo biloba* leaves (GBL) contain a wealth of bioactive compounds, such as flavonoids, terpenoids, and organic acids, which play a significant role in their various therapeutic uses (State et al. 2024). GBL extracts are important for preventing and treating a variety of health disorders due to their antioxidant, anti‐inflammatory, and neuroprotective properties. Findings emphasize their role in enhancing cognitive function, especially concerning age‐related memory decline (Liu et al. 2024) and neurodegenerative conditions like Alzheimer's disease (Pagotto et al. 2024). Furthermore, GBL are recognized for their ability to improve circulation, which may provide advantages for cardiovascular health, particularly in the management of ischemic heart disease (Chen et al. 2024).

*Astragalus membranaceus* (AM), an important herb in traditional Chinese medicine, is valued for its wide range of therapeutic benefits. Saponins (astragalosides), flavonoids, and polysaccharides in these roots contribute significantly to its immunomodulatory, antioxidant, and anti‐inflammatory activities (Klichkhanov and Suleimanova 2024). AM is well‐known for its role in enhancing immune function, contributing to the body's defense against infections and supporting overall vitality. Furthermore, investigations have highlighted its advantages for cardiovascular health, especially in enhancing circulation, lowering blood pressure, and safeguarding against heart disease (Cao et al. 2024; Netala et al. 2024). AM demonstrates potential in the management of chronic conditions like diabetes, renal disease, and cancer, attributed to its capacity to regulate oxidative stress and inflammation (He et al. 2025; Li et al. 2024).

*Salvia miltiorrhiza* (SM) is a traditional Chinese medicinal herb rich in bioactive compounds, notably phenolic acids (e.g., salvianolic acid) and tanshinones. These constituents exhibit strong antioxidant, anti‐inflammatory, and cardioprotective effects. Studies confirm their role in improving blood circulation, reducing oxidative stress, and managing cardiovascular conditions such as angina, hypertension, and ischemic heart disease. Additionally, SM has shown therapeutic potential in treating liver disorders, inflammatory diseases, and certain cancers (Wang et al. 2024; Li et al. 2024; Tavares et al. 2025; Yang et al. 2025).

Methods such as maceration, decoction, and Soxhlet extraction have been used for ages to isolate bioactive chemicals from medicinal plants. These procedures are successful but need a lot of solvent, energy, and time, which might harm sensitive compounds and the environment (Bhadange et al. 2024). Recent advancements in green extraction techniques, including ultrasound‐assisted extraction (UAE), supercritical fluid extraction (SFE), pulsed electric field (PEF), cold plasma, and microwave‐assisted extraction (MAE), present more sustainable alternatives (Alkanan et al. 2024; Cannavacciuolo et al. 2024; Pankaj et al. 2025). These methods reduce solvent use, energy use, and processing time while boosting yield and bioactive component integrity (Morón‐Ortiz et al. 2024). Encapsulation strategies, including spray‐drying and freeze‐drying, can further improve the stability, delivery, sensory acceptability, and food applicability of phenolic‐rich extracts (Pashazadeh et al. 2021). Although these plants have been individually studied, the synergistic interactions of their blends and their impact on extraction efficiency, bioactivity, and digestion stability remain underexplored. Phenolic‐rich extracts may also have multifunctional potential beyond foods, including cosmetic applications such as antioxidant or UV‐supporting ingredients. Nevertheless, regulatory requirements differ substantially according to the intended market and claim. If the extract is proposed as a functional food or supplement ingredient, safety, botanical authentication, contaminant limits, dosage, interaction risks, and claim substantiation must be addressed. Disease‐prevention or therapeutic claims would require a higher level of evidence and may shift the product toward medicinal or botanical‐drug regulation (Galanakis 2018; Galanakis et al. 2018).

To the best of our knowledge, no previous study has optimized the green extraction of a defined tri‐herbal blend composed of *Ginkgo biloba* leaves, *Astragalus membranaceus* roots, and *Salvia miltiorrhiza* roots while simultaneously linking extraction optimization with phytochemical profiling, multiple in vitro bioactivity assays, and simulated gastrointestinal digestion. Most previous studies have examined these plants individually or have focused on extraction yield and antioxidant properties only. In contrast, the present work identifies a synergistic blend ratio, compares three green extraction platforms (probe‐UAE, bath‐UAE, and MAE), validates the optimized extract experimentally, and extends the evaluation to antimicrobial, anti‐inflammatory, cytotoxic, α‐amylase inhibitory, and bioaccessibility outcomes. This integrated approach represents the principal novelty and practical relevance of the study. Therefore, this study addresses this gap by investigating the synergistic effect of blending GBL, AM, and SM and the impact of green extraction methods on this synergistic property.

## 2 Materials and Methods

### 2.1 Materials and Chemicals

Commercially freeze‐dried *Ginkgo biloba* leaves (GBL), *Astragalus membranaceus* roots (AMR), and *Salvia miltiorrhiza* roots (SMR) were obtained from the local market in Zhenjiang, Jiangsu, China; therefore, the lyophilization conditions (temperature, pressure, and drying time) were not controlled and were not disclosed by the supplier. Upon receipt, the samples were kept in airtight containers protected from light until milling and analysis. All analytical grade chemicals and reagents used in this study were purchased from Sigma Chemical Co. Ltd. (St. Louis, MO, USA).

### 2.2 Preparation of Different Blends

Various combinations of GBL, AMR, and SMR were created by mixing the finely powdered plant materials in different ratios. The blends were created by combining plant powders at specific ratios (1:1:1, 2:1:1, 1:2:1, and 1:1:2). The Folin–Ciocalteu assay was conducted to assess the total phenolic content (TPC) of each individual plant and blends. To evaluate if the measured phenolic content for each blend surpassed, equaled, or was less than the anticipated additive contribution of its individual components, a “sum‐of‐parts” calculation was employed (Da Silva Pereira et al. 2015). The predicted TPC for each blend was determined by calculating the weighted average of the single‐extract TPC values, reflecting the proportion of each component in the mixture. To determine TPC‐based apparent synergy, the observed TPC to predicted TPC ratio (Synergy Index) was utilized to discern potential synergistic (> 1), additive (=1), or antagonistic (< 1) outcomes.

$$\begin{array}{cc} {\mathrm{TPC}}_{\text{predicted}}\;=\; & \left[{W_{\mathrm{GBL}}}^{*}\left({\mathrm{TPC}}_{\mathrm{GBL}}\right)\right]+\left[{W_{\mathrm{AMR}}}^{*}\left({\mathrm{TPC}}_{\mathrm{AMR}}\right)\right]\\ & +\left[{W_{\mathrm{SMR}}}^{*}\left({\mathrm{TPC}}_{\mathrm{SMR}}\right)\right] \end{array}$$

$$\text{Synergy Index}=\frac{{\mathrm{TPC}}_{\text{observed}}}{{\mathrm{TPC}}_{\text{predicted}}}$$

The mixtures were subsequently kept in sealed containers at ambient temperature, shielded from direct sunlight, until they were needed for further analysis.

### 2.3 Maceration Extraction Method

GBL, AMR, SMR, and their blends were processed in an analytical mill to achieve a particle size of 1 mm (Cole‐Parmer, Vernon Hills, IL, USA), then sieved to a 50 mesh, and subsequently extracted at room temperature with ethanol at a concentration of 70% (Šalić et al. 2024; Tian et al. 2024; Xu et al. 2025). About 2 g of the powdered sample was subjected to maceration in 50 mL of 70% ethanol and agitated for 24 h in dark glass containers to shield against light exposure. The solution was subsequently subjected to filtration employing Whatman No. 1 filter paper. The filtrate was preserved at −18°C for subsequent analysis.

### 2.4 Green Extraction Methods

The solid‐solvent mixture (1:25 solid‐to‐solvent ratio) was prepared for green extraction using a high‐speed homogenizer (Model: 400ELPC, PRO Scientific Inc., 01‐02411ELPC HOMOGENIZER, Oxford, CT, USA) at 10,000 rpm for 5 min (Yang et al. 2021).

#### 2.4.1 Probe Ultrasound‐Assisted Extraction

The probe ultrasound‐assisted extraction (PUAE) of the prevailing synergistic blend (PSB) was carried out using a 750 W ultrasonic processor (Sonics & Materials Inc., VCX750 Model, Newtown, CT, USA) featuring a 0.5‐in. probe and functioning at a frequency of 20 kHz. Sonication was performed at 25°C using 10‐s pulse intervals. To mitigate the risk of overheating, the beakers were positioned in an ice bath throughout the procedure (Abedelmaksoud et al. 2022).

#### 2.4.2 Bath Ultrasound‐Assisted Extraction

Bath ultrasound‐assisted extraction (BUAE) of PSB was performed using a bath ultrasonic system (Model: FSF‐020S, Faithful Instrument Co. Ltd., China) operating at a fixed frequency of 40 kHz and an output power of 120 W (Vinatoru 2001; Shen et al. 2023).

#### 2.4.3 Microwave‐Assisted Extraction

Microwave‐assisted extraction (MAE) of PSB was performed in a mechanically modified microwave oven (ETHOS 1600, Milestone, Sorisole, Italy) at 40°C. After filtration and cooling to room temperature, the extract was collected and kept at −20°C until analysis (Bagade and Patil 2021; Mokaizh et al. 2024).

### 2.5 Experimental Design

Response Surface Methodology (RSM) was employed to optimize and investigate the effects of all green extraction conditions (Table S1) on polyphenolic extraction yield. A 2‐factor central composite design (CCD) with 13 experimental runs (Table S2) derived from Design‐Expert version 11.1.2.0 (Statease Inc., Minneapolis, MN 55413, USA). Regarding the PUAE, the selected levels of sonication power (*X*_1_) and sonication time (*X*_2_) were (100–400 W) and (10–60 min) while for BUAE, the selected levels of sonication time (*X*_1_) and temperature (*X*_2_) were (5–45 min) and (30°C–60°C). For MAE, the selected levels of extraction time (*X*_1_) and microwave power (*X*_2_) were (5–32.5 min) and (100–900 W). Analysis of variance (ANOVA) was performed to determine the statistical significance of model terms, and model adequacy was assessed through ANOVA statistics. Regression coefficients were used to develop response surface plots, which provided insights into factor interactions and their impact on phenolic extraction yield.

The experimental data were fitted to a second‐order polynomial model:

$$Y=a_{o}+a_{1}\;X_{1}+a_{2}\;X_{2}+a_{12}\;X_{1}\;X_{2}+a_{11}\;{X_{1}}^{2}+a_{22}\;{X_{2}}^{2}$$

where *Y* is the expected response of total phenolic content (TPC), *a*_o_ the estimated regression coefficient of the fitted response at the central point of the model, *a*_1_, *a*_2_ the coefficient of regression for linear effect expressions, *a*_11_, *a*_22_ the quadratic effects, and *a*_12_ the effects of interaction.

Evaluation of model adequacy relied on *R* ^2^ and adjusted *R* ^2^ values, ideally exceeding 0.90, and Prediction Error Sum of Squares (PRESS). A strong model fit was suggested by a high predicted *R* ^2^ and low PRESS value. In addition, perturbation plots showed each factor's design space impact. To determine the optimal conditions for maximizing TPC yield, the desirability function approach was applied, following the methodology described by Abedelmaksoud et al. (2018). Response surface and contour plots were generated to illustrate factor interactions and their effects on total phenolic content yield.

### 2.6 Chemical Composition

For each sample, the analysis of crude fat and ash content were determined according to (“Off. Methods Anal. AOAC Int.,” 2019). The evaluation of crude protein content was conducted using Kjeldahl's method following the procedure of Liu et al. (2023). Finaly, total carbohydrates content was calculated by difference.

### 2.7 Total Phenolic Content and Phenolic Fractions by HPLC

The extracts' TPC was assessed through the Folin–Ciocalteu assay and is reported as mg of gallic acid equivalents (GAE) per gram of powder, following the method outlined (Singleton and Rossi Jr 1965; Pérez et al. 2023). Phenolic compounds were identified using high performance liquid chromatography (HPLC) following the protocol described by Emam et al. (2024). HPLC analysis was conducted utilizing an Agilent 1260 series. The separation utilized a Zorbax Eclipse Plus C8 column with dimensions of 4.6 mm × 250 mm and a particle size of 5 μm. The mobile phase was composed of water (A) and 0.05% trifluoroacetic acid in acetonitrile (B), with a flow rate of 0.9 mL/min. The mobile phase was systematically programmed in a linear gradient as outlined: 0 min (82% A); 0–1 min (82% A); 1–11 min (75% A); 11–18 min (60% A); 18–22 min (82% A); 22–24 min (82% A). The multi‐wavelength detector underwent monitoring at 280 nm. The volume of injection utilized for each of the sample solutions was 5 μL. The temperature of the column was held steady at 40°C.

### 2.8 Total Flavonoids

The modified AlCl_3_ calorimetric method was employed (Chang et al. 2002; Sembiring et al. 2018), with 1 mL of extract dissolved in 2 mL of methanol. Two hundred microliters of extract were mixed with 75 μL of 5% NaNO_3_, and the mixture was allowed to stand for 5 min at room temperature. Subsequently, 1.25 mL of 7% AlCl_3_ and 0.5 mL of 5% NaOH were sonicated and then incubated for 5 min at room temperature.

The absorbance was assessed using methanol as a blank at 510 nm. The results are expressed as micrograms of quercetin equivalent per 1 g of dry extract.

### 2.9 Total Alkaloids

Total alkaloid content was determined by the bromocresol green (BCG) complexation method described by Shamsa et al. (2008). Briefly, 1 mL of extract solution (1 mg/mL) was mixed with 5 mL phosphate buffer (pH 4.7) and 5 mL BCG solution. The mixture was extracted vigorously with 4 mL chloroform, the chloroform layer was collected, and the final volume was adjusted to 10 mL with chloroform. Absorbance was measured at 470 nm against a reagent blank. Atropine was used as the reference standard, and total alkaloid content was expressed as atropine equivalents per g extract.

### 2.10 Total Tannins

Tannin content was determined by the acidified vanillin method according to Broadhurst and Jones (1978), with slight modifications. Briefly, the plant extract was reacted with vanillin reagent under strongly acidic conditions, and the resulting red‐colored complex was measured spectrophotometrically at 500 nm. Quantification was performed using catechin as the reference standard, and the results were expressed as catechin equivalents. The assay is based on the reaction of vanillin with flavan‐3‐ol units of condensed tannins (proanthocyanidins), providing a colorimetric estimate of condensed tannin concentration (Sadeghi et al. 2024; Li et al. 2023).

### 2.11 Antioxidant Activity

*1, 1‐diphenyl‐2‐picryl hydrazyl (DPPH)* was used to assess the free radical scavenging activity of prevailing synergistic extract (PSE) according to the protocol described by González‐Palma et al. (2016). The reaction mixture consisted of 0.5 mL of extract, 3 mL methanol, and 0.3 mL of 0.5 mM DPPH solution in methanol. After incubation for 45 min in the dark at room temperature, absorbance was measured at 517 nm. Ascorbic acid was used as the positive control. The percent DPPH scavenging effect was calculated by using the following equation:

$$\text{DPPH Scavenging}\;\left(\%\right)=\frac{A_{0}-A_{1}}{A_{0}}*100$$

where *A*_0_ was the absorbance of the control reaction and *A*_1_ was the absorbance in the presence of the test or standard sample. IC_50_ values were estimated from the concentration–response curve.

*ABTS radical scavenging activity* was assessed following the methodology outlined by González‐Palma et al. (2016), with certain modifications implemented. ABTS was dissolved in water to achieve a concentration of 7 mM. The 2,20‐azino‐bis(3‐ethylbenzothiazoline‐6‐sulphonicacid) (ABTS) radical cation (ABTS^+^) was generated by combining the ABTS stock solution with a 2.45 mM potassium persulfate solution (final concentration) and permitting the mixture to rest in the dark at room temperature for a duration of 12–16 h prior to application. The ABTS^+^ solution was diluted with water to achieve an absorbance of 0.70 at 734 nm. The reaction mixture included 0.07 mL of extract combined with 3 mL of the ABTS radical. Following a 6‐min incubation period, the absorbance was measured using a spectrophotometer at a wavelength of 734 nm. The antioxidant activity was calculated by using the following equation:

$$\%\text{Inhibition}=\frac{A_{\text{control}}-A_{\text{sample}}}{A_{\text{control}}}*100$$

where *A*_control_ was the absorbance of the negative control at the moment of solution preparation and *A*_sample_ was the absorbance of the sample after 6 min.

The EC_50_ values were derived from the graph illustrating the concentration of the sample necessary to neutralize 50% of the ABTS free radicals. The EC_50_ value is commonly utilized to indicate the quantity or concentration of extracts required to neutralize 50% of the free radicals. ABTS is quantified in micrograms of gallic acid equivalents per milliliter.

*Ferric reducing antioxidant power (FRAP)* was used to investigate the impact of solvent polarity on the total reducing power of PSE, potassium ferricyanide, trichloroacetic acid method was used (Benzie and Strain 1996) with some modifications and adaptation for the microplate method (Athamena et al. 2019). Eppendorf tubes were labeled, and a 40 mL sample was added to each tube. This was followed by the addition of 50 mL of sodium phosphate dihydrate (Na_2_HPO_4_.2H_2_O) buffer at a concentration of 0.2 mol/L, 50 mL of 1% potassium ferricyanide (K_3_Fe(CN)_6_), and 50 mL of 10% trichloroacetic acid. The sample underwent centrifugation at 3000 rpm for a duration of 10 min. Following centrifugation, 166.66 mL of the supernatant from each sample was transferred to 96 well plates, and 33.3 mL of ferric chloride (FeCl_3_, 1%) was subsequently added. The measurements were conducted at 630 nm using a microtiter plate reader (Biotek ELX800; Biotek, Winooski, VT, USA). DMSO was used as a negative control and ascorbic acid (1 mg/mL) as a positive control. Results were expressed as ascorbic acid equivalent (AAE) mg/mg of extract.

*Total antioxidant capacity (TAC) of* PSE was assessed spectrophotometrically by the phosphomolybdenum method according to the procedure described by Prieto et al. (1999). This assay is based on the reduction of Mo(VI) to Mo(V) by antioxidants in the sample and the subsequent formation of a green phosphate/Mo(V) complex in acidic medium. The reaction mixture was incubated at 95°C for 90 min, cooled to room temperature, and the absorbance was measured at 695 nm. Values were reported as ascorbic acid equivalent (AAE) μg/mg of extracts (Lahmass et al. 2018; Chatepa et al. 2024).

### 2.12 Antimicrobial Activity

The agar well diffusion method was utilized to assess the antimicrobial activity of PSE, adhering to the Clinical and Laboratory Standards Institute (CLSI) guidelines (Nagpal et al. 2024). The test microorganisms were cultivated on suitable growth media, utilizing Mueller‐Hinton Agar (MHA, pH 7.2–7.4) for bacterial strains and Sabouraud Dextrose Agar (SDA) for fungal strains to promote optimal microbial growth. A standardized inoculum suspension was created utilizing the direct colony suspension method and calibrated to the 0.5 McFarland standard (1 × 10^8^ CFU/mL). Using a sterile swab, the inoculum was evenly spread across the agar surface in three directions. A sterile cork borer was used to carefully make 6 mm wells after the agar surface dried for 15 min. Each well contained 100 μL of the sample solution at the specified concentration. Plates were left undisturbed for a brief time to allow antimicrobial agent diffusion, then incubated at 35°C for 16–24 h for bacteria and 28°C–30°C for 48 h for fungi. The zone of inhibition (mm) was measured with a calibrated ruler in the area where microbial growth decreased significantly (Espinel‐Ingroff et al. 2007, 2011; Magaldi et al. 2004).

### 2.13 Minimal Inhibitory Concentration (MIC)

The minimal inhibitory concentration (MIC) of PSE was determined against *Bacillus subtilis* (ATCC 6633), *Staphylococcus aureus* (ATCC 6538), *Klebsiella pneumoniae* (ATCC 13883), *Salmonella typhimurium* (ATCC 6539), *Candida albicans* (ATCC 10221), and *Aspergillus niger* (ATCC 16888) using the broth microdilution method following ISO 20776‐1 guidelines (Wikler et al. 2006). A stock solution (1000 μg/mL) was prepared by dissolving 10 mg of PSE in 10 mL distilled water. Serial twofold dilutions (1000–1.95 μg/mL) were made in Tryptic Soy Broth within 96‐well microtiter plates. Negative controls (broth only) and positive controls (broth with inoculum but no antimicrobial agent) were included. The microbial inoculum was adjusted to 0.5 McFarland standard (1 × 10^8^ CFU/mL), then diluted to 5 × 10^5^ CFU/mL per well. Inoculation volume was limited to ≤ 10 μL in each 100 μL well. Plates were incubated at 35°C for 16–20 h in ambient air. MIC values were defined as the lowest concentration showing no visible growth, confirmed by turbidity assessment and optical density (OD) measurement at 630 nm using a BioTek 800 TS microplate reader. Results were compared with controls to ensure accurate MIC determination.

### 2.14 Anti‐Inflammatory

In order to evaluate the anti‐inflammatory activity (Ameena et al. 2023), a volume of 50 μL of PSE was utilized, with a series of concentrations including 1.56, 3.12, 6.25, 12.5, 25, 50, 100, and 200 μg/mL being incorporated into 450 μL of a 1% aqueous solution of bovine serum albumin. The solution's pH was adjusted to 6.3 by adding a precise quantity of 1N hydrochloric acid. The samples underwent incubation at room temperature for a duration of 20 min, and were subsequently subjected to heating at 55°C for 30 min in a water bath. Following the heating process, the samples were permitted to cool, and the absorbance was assessed using a Biosystem 310 plus spectrophotometer at a wavelength of 670 nm. Diclofenac sodium served as the reference medication for comparison purposes. In this experiment, dimethyl sulfoxide (DMSO) served as the control substance.

The percentage of protein denaturation was determined using the following equation:

$$\%\text{Inhibition}=\frac{{\mathrm{Abs}}_{\text{control}}-{\mathrm{Abs}}_{\text{sample}}}{{\mathrm{Abs}}_{\text{control}}}*100$$

where Abs_control_ was the absorbance of control and Abs_sample_ was the absorbance of sample.

### 2.15 Cytotoxicity

The reduction of yellow MTT (3‐(4,5‐dimethylthiazol‐2‐yl)‐2,5‐diphenyl tetrazolium bromide) to purple is dependent on mitochondrial activity. Formazan was utilized to assess cell viability (Hashem et al. 2023). All procedures were conducted in a sterile environment utilizing a biosafety class II Laminar flow cabinet (Baker, SG403INT, Sanford, ME, USA). Cells were suspended in DMEM‐F12 medium along with a normal cell line (BJ1), supplemented with a 1% antibiotic‐antimycotic mixture (10,000 U/mL potassium penicillin, 10,000 μg/mL streptomycin sulfate, and 25 μg/mL amphotericin B) and 1% L‐glutamine, maintained at 37°C in a 5% CO_2_ environment. Cells were cultured in batches for a duration of 10 days, subsequently seeded at a concentration of 10 × 10^3^ cells per well in fresh complete growth medium within 96‐well microtiter plastic plates. The incubation was conducted at 37°C for 24 h under 5% CO_2_, utilizing a water‐jacketed carbon dioxide incubator (Sheldon, TC2323, Cornelius, OR, USA). The media was aspirated, fresh medium (serum‐free) was introduced, and the cells were incubated either in isolation (negative control) or with varying concentrations of PSE to achieve final concentrations of 100, 50, 25, 12.5, 6.25, 3.125, 0.78, and 1.56 μg/mL. Following a 48‐h incubation period, the medium was removed, and 40 μL of MTT salt (2.5 μg/mL) was introduced to each well. The samples were then incubated for an additional 4 h at 37°C in a 5% CO_2_ environment. To halt the reaction and dissolve the resulting crystals, 200 μL of 10% sodium dodecyl sulfate (SDS) in deionized water was introduced to each well and incubated overnight at 37°C. A positive control consisting of 100 μg/mL was utilized as a recognized cytotoxic natural agent that achieves 100% lethality under identical conditions. The absorbance was subsequently assessed utilizing a microplate multi‐well reader (Bio‐Rad Laboratories Inc., model 3350, Hercules, CA, USA) at wavelengths of 595 and 620 nm as a reference point. Statistical differences among treatment concentrations and the negative control were evaluated using one‐way ANOVA followed by Dunnett's post hoc test at *p* < 0.05. The solvent employed for dissolving plant extracts was DMSO, with a final concentration on the cells remaining below 0.2%. According to the following formula, the percentage of change in viability was calculated:

$$\%\text{Change in viability}=\left(\frac{{\text{Reading}}_{\text{extract}}}{{\text{Reading}}_{\text{negative control}}}-1\right)*100$$

IC_50_ and IC_90_ were computed using probit analysis. The selectivity of synthetic compounds was assessed using the formula SI = IC_50_ of the pure chemical in a normal cell line divided by IC_50_ in a cancer cell line, where IC_50_ is the concentration needed to kill 50% of a cell population.

### 2.16 In Vitro Digestion and Bioaccessibility

The in vitro digestion model was based on the standardized INFOGEST protocol (Brodkorb et al. 2019) with specific modifications according to Rusak et al. (2021) to reflect the physicochemical characteristics of the polyphenol‐rich extract matrix. Initially, a volume of 0.3 mL of PSE was combined with an equal volume of 20 mM phosphate buffer at pH 7.0. The salivary phase of digestion commenced with the addition of 10 μL of amylase (0.48 mg/mL in 20 mM phosphate buffer pH 7.0) and was incubated for 5 min at 37°C in a shaking water bath set to 150 rpm. In order to replicate the process of stomach digestion, 0.4 mL of porcine pepsin solution (3 mg/mL in 0.1 M HCl) was introduced and subsequently acidified using 0.5 M HCl to achieve a pH of 2.0. The samples underwent incubation in a shaking water bath SW22 (Julabo, Seelbach, Germany) for 1 h at 37°C with a speed of 150 rpm. The initial phase of upper intestinal digestion was replicated by incorporating sodium bicarbonate (1 M NaHCO_3_) to modify the pH to 5.3. Following the adjustment of pH, a volume of 0.9 mL of pancreatic juices, containing 2.4 mg bile acids/mL, 0.2 mg porcine lipase/mL, and 0.4 mg pancreatin/mL in a 20 mM phosphate buffer at pH 7.0, was introduced. The final total volume of each intestinal phase sample was adjusted to 2 mL using a 20 mM phosphate buffer at pH 7.0. The final pH was adjusted to 7.0 using 1 M NaOH. The samples underwent incubation for 2 h at 37°C in a shaking water bath set to 150 rpm. The final volume of each sample, prior to and following digestion, was adjusted to 2 mL using 20 mM phosphate buffer (pH 7.0). The samples underwent centrifugation using a Hettich MIKRO 220R (Andreas Hettich GmbH & Co., Tuttlingen, Germany) at 11,000 rpm for 10 min at a temperature of 4°C. The resulting supernatants were subsequently stored at −20°C in preparation for spectrophotometric. The in vitro digestion was conducted in triplicate.

The bioaccessibility of PSE bioactive compounds (BC) was calculated from the following equations:

$$\text{Bioaccessibility in salivary phase}\;\left(\%\right)=\frac{{\mathrm{BC}}_{\text{salivary phase}}}{{\mathrm{BC}}_{\text{before digestion}}}*100$$

$$\text{Bioaccessibility in gastric phase}\;\left(\%\right)=\frac{{\mathrm{BC}}_{\text{gastric phase}}}{{\mathrm{BC}}_{\text{before digestion}}}*100$$

$$\text{Bioaccessibility in intestinal phase}\;\left(\%\right)=\frac{{\mathrm{BC}}_{\text{intestinal phase}}}{{\mathrm{BC}}_{\text{before digestion}}}*100$$

The BC_salivary phase_, BC_gastric phase_, and BC_intestinal phase_ were associated with the total or individual concentrations of bioactive compounds in the salivary, gastric, and intestinal phases, respectively, while BC_before digestion_ represented the total or individual concentrations of bioactive compounds prior to in vitro digestion.

### 2.17 In Vitro α‐Amylase Inhibition (Antidiabetic Effect)

The assay for α‐amylase inhibition was conducted utilizing the 3,5‐dinitrosalicylic acid (DNSA) method (Wickramaratne et al. 2016). PSE was initially dissolved in a minimal volume of 10% DMSO and subsequently diluted in a buffer solution composed of Na_2_HPO_4_/NaH_2_PO_4_ (0.02 M) and NaCl (0.006 M) at pH 6.9, resulting in concentrations varying from 1.9 to 1000 μg/mL. Two hundred microliters of α‐amylase solution (2 units/mL) were combined with 200 μL of the extract and incubated for 10 min at 30°C. Subsequently, 200 μL of the starch solution (1% in water (w/v)) was introduced into each tube and allowed to incubate for 3 min. The reaction was halted through the incorporation of 200 μL DNSA reagent, which consists of 12 g of sodium potassium tartrate tetrahydrate dissolved in 8.0 mL of 2 M NaOH and 20 mL of a 96 mM solution of 3,5‐dinitrosalicylic acid. This mixture was then subjected to boiling for 10 min in a water bath maintained at a temperature of 85°C–90°C. The solution was allowed to cool to room temperature and subsequently diluted with 5 mL of distilled water. The absorbance was then recorded at 540 nm utilizing a UV–Visible Biosystem 310 spectrophotometer. The Control with 100% enzyme activity was prepared by substituting the plant extract with 200 μL of buffer. A control reaction was prepared using the plant extract at each concentration without the enzyme solution. The α‐amylase inhibitory activity was expressed as percent inhibition and was calculated using the equation given below:

$$\%\alpha -\text{amylase inhibition}=\frac{{\mathrm{Abs}}_{\text{control}}-{\mathrm{Abs}}_{\text{sample}}}{{\mathrm{Abs}}_{\text{control}}}*100$$

The % α‐amylase inhibition was plotted against the extract concentration and the IC_50_ values were obtained from the graph.

### 2.18 Statistical Analysis

This study employed SPSS 11 (SPSS, Chicago, IL, USA) for technical triplicate experiments. For comparisons among more than two groups, one‐way ANOVA was used followed by Tukey's HSD post hoc test when the ANOVA was significant (*p* < 0.05). For comparisons of multiple concentrations against a single untreated control (e.g., cytotoxicity or anti‐inflammatory assays), one‐way ANOVA followed by Dunnett's post hoc test was used. IC_50_/EC_50_ values were calculated by nonlinear regression of concentration–response curves. For RSM, model significance and adequacy were evaluated in Design‐Expert by ANOVA, including model *F*‐value, *p*‐value, lack‐of‐fit test, *R* ^2^, adjusted *R* ^2^, predicted *R* ^2^, PRESS, and adequate precision. Exact *F* values, degrees of freedom, exact *p* values, and sample size (*n*) are reported in the Results, tables, and figure legends. Statistical significance was set at *p* < 0.05.

## 3 Results and Discussion

### 3.1 Synergistic Blend

The sum‐of‐parts analysis according to Table 1, indicated different levels of TPC‐based apparent synergy and antagonism in total phenolic content across the tested blends. The 1:1:2 blend of *Ginkgo biloba* leaves (GBL), *Astragalus membranaceus* roots (AMR), and *Salvia miltiorrhiza* roots (SMR) demonstrated a 34% increase compared to the predicted additive total phenolic content (TPC), suggesting a synergistic interaction that could improve phenolic extraction or stability and making it the prevailing synergistic blend (PSB) and prevailing synergistic extract (PSE). The 1:2:1 mixture had a 22% lower TPC than expected, showing a negative interaction that reduced phenolic concentration. These findings demonstrate the importance of mixture composition in maximizing phenolic yield and show that certain ratios may extract or stabilize phenolic chemicals more efficiently. Additional bioactivity experiments are needed to convert these chemical synergy indications into functional or therapeutic advantages.

**TABLE 1:** Interactions caused by different blending ratios.

Plant	Observed (mg GAE/g)	Predicted (mg GAE/g)	Synergy Index (Observed/Predicted)	Interpretation
GBL	168.56^e^	—	—	—
AMR	114.66^g^	—	—	—
SMR	197.96^b^	—	—	—
1:1:1 blend	183.26^c^	160.39	1.14	Synergy (+14%)
2:1:1 blend	178.36^d^	162.44	1.10	Synergy (+10%)
1:2:1 blend	115.66^f^	148.96	0.78	Antagonism (−22%)
1:1:2 blend	227.36^a^	169.79	1.34	Synergy (+34%)

*Note:* Values with different lowercase superscript letters are significantly different according to one‐way ANOVA followed by Tukey's HSD test at *p* < 0.05. Predicted values and synergy index were calculated using the sum‐of‐parts model.

It should be noted that the synergy index used here provides a first‐level estimate of interaction based on total phenolic recovery, rather than definitive proof of molecular synergy among individual phytochemicals. The observed enhancement may result from improved solvent penetration, co‐extraction effects, protection of phenolics from degradation, or matrix‐mediated stabilization. Therefore, further work should fractionate the extract, test individual and recombined fractions, and apply quantitative interaction models such as isobolographic analysis, Bliss independence, Loewe additivity, or Chou–Talalay combination index analysis to distinguish additive, synergistic, and antagonistic interactions across concentrations and bioactivities.

### 3.2 Optimized Green Extraction

The regression models derived from Response Surface Methodology (RSM) for the three green extraction techniques (probe ultrasonication, bath ultrasonication, and microwave‐assisted extraction) were fitted to a second‐order polynomial equation, illustrating the impact of extraction conditions (Table S1) on phenolic yield (mg GAE/g). The equations facilitated the prediction of extraction outcomes and clarified the influence of individual, interaction, and quadratic terms on the phenolic recovery from the synergistic blend of GBL, AMR, and SMR (1:1:2).

Regression model for probe ultrasound‐assisted extraction yield (*Y*_1_):

$$Y_{1}=333.21+11.14X_{1}+6.42X_{2}-0.6030X_{1}X_{2}-1.64{X_{1}}^{2}-1.26{X_{2}}^{2}$$

The model indicates that both sonication power (*X*_1_) and time (*X*_2_) have a positive impact on phenolic yield, as evidenced by their positive linear coefficients (+11.14 and +6.42, respectively). This indicates that augmenting either parameter within the examined range improves the extraction efficiency. The interaction term (*X*_1_ *X*_2_ = −0.6030) and the negative quadratic coefficients (*X*_1_^2^ = −1.64; *X*_2_^2^ = −1.26) indicate a concave downward trend, suggesting that while yield increases with sonication power and time, the effect levels off or may even decrease at elevated levels. This behavior is commonly observed in ultrasonic‐assisted extraction, where excessive processing may result in the degradation of compounds or saturation of the solvent system. The superior performance of ultrasound‐assisted extraction observed in the present study is consistent with the findings of Okumus (2024), who reported that ultrasonic extraction of *Crambe tataria* yielded higher TPC and stronger antioxidant and antidiabetic activity than conventional extraction, particularly in flower samples. Although the botanical matrix differs, both studies support the role of acoustic cavitation in enhancing solvent penetration, cell‐wall disruption, and mass transfer. In summary, probe ultrasonication exhibited the greatest yield and the most advantageous response surface curvature, suggesting a clearly defined optimal area for enhancing recovery.

Regression model for bath ultrasound‐assisted extraction yield (*Y*_2_):

$$Y_{2}=264.11+9.37X_{1}+13.76X_{2}+0.9233X_{1}X_{2}+1.97{X_{1}}^{2}-0.0900{X_{2}}^{2}$$

In contrast, bath ultrasonication demonstrated a more significant influence of extraction temperature (*X*_2_ = +13.76) relative to time (*X*_1_ = +9.37). Notably, the quadratic term for time (*X*_1_^2^ = +1.97) was positive, suggesting a convex trend, which could indicate an increase in phenolic yield at extended durations beyond the midpoint of the experimental design. Nevertheless, the temperature squared term (*X*_2_^2^ = −0.0900) approached zero, indicating a predominantly linear response to temperature variations. The positive interaction term (*X*_1_ *X*_2_ = +0.9233) indicates that time and temperature together contribute to an increase in extraction, though to a modest extent. The results indicate that bath ultrasonication necessitates more precise management of duration, as extended exposure could alter yield patterns in a non‐linear manner.

Regression model for microwave‐assisted extraction yield (*Y*_3_):

$$Y_{3}=207.02-20.66X_{1}-13.87X_{2}+8.91X_{1}X_{2}-23.21{X_{1}}^{2}-19.43{X_{2}}^{2}$$

The regression equation for microwave‐assisted extraction showed negative linear coefficients for both time (*X*_1_ = −20.66) and power (*X*_2_ = −13.87), suggesting that elevating either parameter beyond specific thresholds negatively impacts phenolic recovery. The significant negative quadratic terms (*X*_1_^2^ = −23.21; *X*_2_^2^ = −19.43) reinforce the presence of a pronounced concave curvature, indicating that both parameters require precise adjustment within a limited optimal range to prevent thermal degradation or ineffective energy distribution. The notable positive interaction term (*X*_1_ *X*_2_ = +8.91) suggests that moderate levels of both variables can create synergistic effects, momentarily counteracting the negative trend. Nonetheless, the comprehensive model indicates the susceptibility of microwave‐assisted extraction to excessive processing, underscoring the necessity for meticulous optimization.

Table S3 presents a summary of the statistical parameters utilized to evaluate the predictive performance of the RSM models created for assessing the phenolic extraction efficiency of three green extraction techniques: probe ultrasonication, bath ultrasonication, and microwave‐assisted extraction. The standard deviation values (0.3526 for probe ultrasonication, 2.13 for bath ultrasonication, and 0.8456 for microwave‐assisted extraction) underscore the variability in the experimental responses. Notably, probe ultrasonication exhibits the least variation, suggesting a high level of repeatability in the data collected. The mean values obtained were 332.28, 264.88, and 192.21 mg GAE/g, respectively, demonstrating that probe ultrasonication produced the highest total phenolic content, with bath and microwave methods following in that order. The coefficient of variation (CV %), serving as a relative measure of precision, was notably low for both probe ultrasonication (0.1061%) and microwave‐assisted extraction (0.44%), significantly under the acceptable threshold of 10%. This finding underscores the high precision and reproducibility of the experimental designs employed. The models' predictive performance underwent additional evaluation through the application of the predicted residual sum of squares (PRESS). The PRESS value for probe ultrasonication was the lowest at 3.05, followed by microwave‐assisted extraction at 14.59. In contrast, bath ultrasonication exhibited a significantly higher PRESS of 158.36, suggesting a weaker agreement between the observed and predicted values for this method. The coefficient of determination (*R* ^2^) values were notably high for probe ultrasonication (0.9991) and microwave‐assisted extraction (0.9990), whereas bath ultrasonication exhibited a slightly lower value (0.9808), which remains within a robust predictive range. The adjusted *R* ^2^ values, which consider the number of predictors in the model, were notably high as 0.9985, 0.9983, and 0.9671 for probe, microwave, and bath ultrasonication, respectively demonstrating the strength of the models.

Additionally, the predicted *R* ^2^ values, which assess the models' ability to predict future results, were 0.9969 (probe), 0.9971 (microwave), and 0.9039 (bath). The findings indicate a remarkably high level of predictability for both probe and microwave‐assisted extraction methods. The precision values, which serve to quantify the signal‐to‐noise ratio, exceeded the acceptable threshold of 4 across all methods: 127.66 (probe), 90.35 (microwave), and 28.79 (bath). The elevated values indicate that the models yield sufficient signals and demonstrate reliability in traversing the design space and fine‐tuning extraction conditions. Table S4 demonstrates that for the bath‐UAE model, the lack‐of‐fit was not significant (*F* = 3.16, *p* = 0.2576), and for the microwave‐assisted extraction model it was also not significant (*F* = 3.52, *p* = 0.1646), indicating adequate model fit. For the probe ultrasonication model, replicated points gave essentially identical responses, resulting in zero pure error, so a formal lack‐of‐fit *F*‐test could not be meaningfully estimated from the dataset.

**FIGURE 1:**
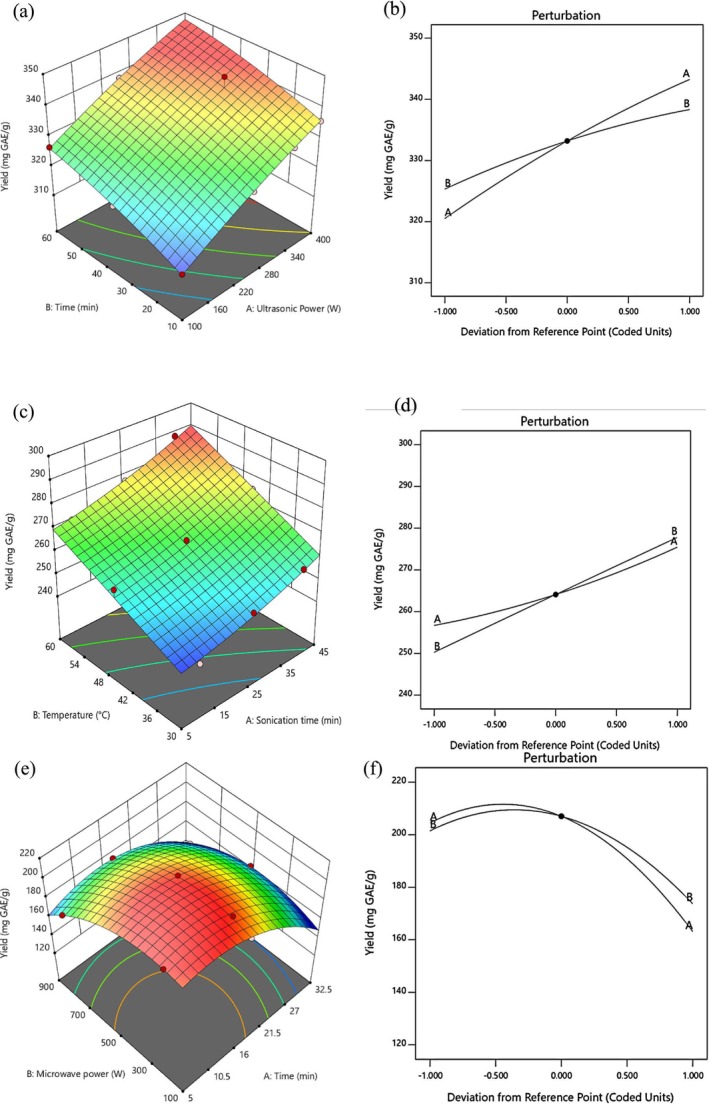
Effect of ultrasonic power and time on PSE: (a, b) 3D surface plot and (b) perturbation plot; Sonication time and temperature on PSE: (c) 3D surface plot and (d) perturbation plot; Time and microwave power on PSE: (e) 3D surface plot and (f) perturbation plot. RSM model significance was evaluated by ANOVA in Design‐Expert at *p* < 0.05.

Figure 1 demonstrates the impact of various green extraction parameters on the phenolic yield (mg GAE/g) derived from the synergistic herbal blend consisting of GBL, AMR, and SMR, utilizing probe ultrasonication, bath ultrasonication, and microwave‐assisted extraction methods. The 3D surface plots and perturbation curves produced from the RSM models offered significant insights into the effects and interactions of variables on extraction performance. The 3D surface plot (Figure 1a) demonstrated a nearly linear positive correlation between ultrasonic power (*X*_1_) and extraction time (*X*_2_) with phenolic yield, achieving a peak of around 342.7 mg GAE/g at elevated settings of both parameters. The surface response exhibited a convex shape, indicating that within the examined range, an increase in power and time consistently improved phenolic recovery without any notable decline, underscoring the lack of degradation effects. The perturbation plot (Figure 1b) reinforces this observation, as both factors display positive slopes, with ultrasonic power (*X*_1_) demonstrating a more significant curvature, suggesting its comparatively greater impact. This indicates that optimizing both variables within feasible constraints would result in the best recovery outcomes. The surface plot for bath ultrasonication (Figure 1c) exhibited a more gradual slope, with the phenolic yield reaching its maximum at approximately 288 mg GAE/g. The influence of temperature (*X*_2_) was notably stronger than that of sonication time (*X*_1_), as evidenced by the steeper gradient observed along the B‐axis. The perturbation plot (Figure 1d) validated this observation, demonstrating that temperature has a more pronounced and linear effect on yield. The response surface's shape indicated a limited interaction between the two factors, and the lack of significant curvature suggested minimal compound degradation within the examined range. Nonetheless, the comparatively lower yield and diminished curvature suggest that bath ultrasonication might be less effective for extracting phenolics from this herbal matrix.

The analysis of microwave‐assisted extraction revealed a clear parabolic response surface (Figure 1e). The phenolic yield showed an initial increase with both time (*X*_1_) and microwave power (*X*_2_), but subsequently decreased after surpassing an optimal threshold, achieving a peak yield of around 210.97 mg GAE/g. This convex surface demonstrates thermal sensitivity, suggesting that extended exposure or excessive power may lead to the degradation of phenolic compounds. The perturbation plot (Figure 1f) highlighted the non‐linear behavior, with both *X*_1_ and *X*_2_ curves reaching their peaks at the center point before sharply declining with additional deviation. The results underscore the critical need for meticulous optimization to prevent thermal degradation in microwave extraction processes. The decline in phenolic yield at higher MAE time and power indicates that microwave energy must be carefully controlled. Excessive energy input may accelerate degradation, oxidation, isomerization, or polymerization of heat‐sensitive phenolic acids and flavonoids. Therefore, the lower MAE yield compared with probe‐UAE may reflect not only extraction efficiency but also partial instability of some phenolics under high‐energy microwave conditions (Sarfarazi et al. 2020).

The results of the optimization for the extraction methods are presented in Table 2, showcasing both the predicted and experimentally observed yields of TPC (measured as mg GAE/g) under the optimal conditions for each technique. The strong alignment between predicted and experimental values across all methods demonstrates the strength of the models used and confirms the dependability of the optimization process. Probe ultrasonication improves phenolic component recovery the most, probably due to its higher energy intensity and direct cavitation effects than bath and microwave methods.

**TABLE 2:** Predicted and observed yield of green extraction methods at optimum conditions.

Method	Optimum conditions		Predicted yield (mg GAE/g)	Observed yield (mg GAE/g)
	*X*_1_	*X*_2_		
Probe‐Ultrasonication	388.414	38.644	343.422	342.73
Bath‐Ultrasonication	44.512	59.877	289.570	281.03
Microwave‐assisted	9.279	186.913	213.989	210.97

*Note:* Probe ultrasonication: sonication power (*X*_1_) and time (*X*_2_); Bath ultrasonication: Time (*X*_1_) and temperature (*X*_2_); Microwave‐assisted: time (*X*_1_) and microwave power (*X*_2_). RSM model significance was evaluated by ANOVA in Design‐Expert at *p* < 0.05. Values represent predicted and experimentally validated optimum TPC yields.

Pressurized hot water extraction (PHWE) represents another relevant green extraction strategy because water under controlled high‐temperature and pressure conditions shows altered polarity and can improve the recovery of several bioactive compounds while reducing the use of organic solvents. PHWE has been successfully applied for the recovery of bioactive compounds and steviol glycosides from *Stevia rebaudiana* leaves, with temperature identified as a critical extraction parameter (Kovačević et al. 2018). Compared with PHWE, probe‐UAE offers strong local cavitation, enhanced solvent penetration, and lower bulk thermal load, which may be advantageous for phenolic‐rich herbal matrices. However, PHWE may offer better process containment and continuous operation potential. Therefore, future work should directly compare probe‐UAE and PHWE for this tri‐herbal blend using equivalent solvent‐to‐solid ratios, energy input, residence time, phenolic profile, antioxidant activity, and cost/scalability indicators.

### 3.3 Proximate Composition

The proximate analysis of GBL, SMR, and AMR indicates unique nutritional compositions that could affect their pharmacological and therapeutic properties (Table 3). Interestingly, GBL showed the highest fat content at 4.61 g/100 g dry weight, which could improve the bioavailability of its lipid‐soluble compounds, thereby contributing to its well‐known antioxidant properties. Regarding mineral content, GBL (9.86 g/100 g dry weight) and SM (9.05 g/100 g dry weight) exhibited elevated ash values in comparison to AM (7.32 g/100 g dry weight). The elevated ash content indicates a more diverse mineral composition in GBL as it contains significant amounts of calcium and potassium (Koczka and Stefanovits‐Banyai 2016) and SMR as it contains large amounts of phosphorus, calcium, magnesium, sulfur, and aluminum (Lv et al. 2019), potentially enhancing their traditional applications in supporting health and wellness. The carbohydrate content was significant in all three species, with SMR exhibiting the highest value at 76.89 g/100 g dry weight, followed closely by GBL at 74.1 g/100 g dry weight and AMR at 73.8 g/100 g dry weight. The notable carbohydrate content, especially as polysaccharides, is recognized for its essential contribution to the medicinal properties of these herbs, frequently linked to immune‐modulating and antioxidant effects. Moreover, AMR exhibited the highest protein content at 15.2 g/100 g dry weight, thereby increasing its nutritional value and potential as a dietary supplement. The results highlight these herbal species' unique nutritional properties, suggesting health benefits and therapeutic potential in traditional and modern medicine.

**TABLE 3:** Main constituents of GBL, SMR, and AMR.

Composition	GBL (g/100 g dw)	SMR (g/100 g dw)	AMR (g/100 g dw)
Fat	4.61 ± 0.12^a^	3.52 ± 0.13^b^	3.68 ± 0.175^b^
Ash	9.86 ± 0.192^a^	9.05 ± 0.162^b^	7.32 ± 0.199^c^
Total carbohydrates	74.1 ± 0.512^b^	76.89 ± 0.491^a^	73.8 ± 0.55^b^
Protein	11.43 ± 0.134^b^	10.54 ± 0.197^c^	15.2 ± 0.186^a^

*Note:* Values are expressed as mean ± SD (*n* = 3). Different lowercase superscript letters within the same row indicate significant differences according to one‐way ANOVA followed by Tukey's HSD test at *p* < 0.05 (Table S5).

Abbreviations: AMR, *Astragalus membranaceus* roots; GBL, *Ginkgo biloba* leaves; SMR, *Salvia miltiorrhiza* roots.

### 3.4 Phytochemicals and Antioxidant Activity

Regarding the identification of phenolic content (Figures S1 and S2), the quantitative analysis of phenolic and flavonoid compounds in PSE demonstrated a varied composition with differing concentrations (Table 4). In the analysis of the detected compounds, rosmarinic acid emerged as the most prevalent, recorded at a concentration of 249.58 μg/mL. This was succeeded by chlorogenic acid at 135.59 μg/mL and catechin at 36.93 μg/mL. Rosmarinic acid, a notable polyphenol, is acknowledged for its strong antioxidant, anti‐inflammatory, and neuroprotective characteristics, positioning it as a significant bioactive compound for prospective therapeutic uses, especially in the prevention of neurodegenerative diseases and the modulation of immune responses (Noor et al. 2022). Chlorogenic acid, a significant hydroxycinnamic acid, demonstrates antioxidant, antidiabetic, and cardioprotective properties. It has been associated with enhanced glucose metabolism and lipid regulation, highlighting its importance in the realm of metabolic disorders (Lukitasari et al. 2021). Significantly, gallic acid (30.84 μg/mL), rutin (21.08 μg/mL), and ferulic acid (21.40 μg/mL) were identified in considerable concentrations, suggesting their possible roles in enhancing the extract's antioxidant and bioactive characteristics. In contrast, kaempferol was absent, while cinnamic acid and coumaric acid were identified at low concentrations of 0.40 and 0.89 μg/mL, respectively. The identification of different hydroxycinnamic and hydroxybenzoic acids, including syringic acid, ellagic acid, and vanillin, indicates a sophisticated phenolic composition that could enhance the functional characteristics of the extract. The results are consistent with earlier studies on plant‐derived polyphenols, reinforcing their possible roles as antioxidants, antimicrobials, and in therapeutic applications (El Kantar et al. 2022; Yan et al. 2020).

**TABLE 4:** Identification of the phytochemicals in the prevailing synergistic extract (PSE).

Compound	Concentration (μg/mL)
Gallic acid	30.84
Chlorogenic acid	135.59
Catechin	36.93
Methyl gallate	4.77
Coffeic acid	7.43
Syringic acid	8.74
Rutin	21.08
Ellagic acid	7.60
Coumaric acid	0.89
Vanillin	4.99
Ferulic acid	21.40
Naringenin	17.82
Rosmarinic acid	249.58
Daidzein	6.39
Querectin	3.48
Cinnamic acid	0.40
Kaempferol	0.00
Hesperetin	2.25

*Note:* Values represent HPLC‐quantified phenolic concentrations. No post hoc statistical comparison was applied to this descriptive profiling table.

Prevailing synergistic extract (PSE) demonstrated significant levels of essential phytochemicals, such as total flavonoids, alkaloids, and tannins (Table 5). These compounds are extensively studied for their ability to neutralize free radicals and enhance the extract's overall antioxidant capacity, anti‐inflammatory, and anticancer effects (Cosme et al. 2025; de Lima et al. 2025). The total flavonoid content measured 1.27 mg quercetin equivalents (QuE/mL), highlighting a significant concentration of these antioxidant compounds. Furthermore, the extract demonstrated a total alkaloid content of 5.11 μg/g, indicating a moderate yet significant presence of nitrogenous compounds. The total tannin content was quantified at 10.63 mg tannic acid equivalents (AE/g), representing the highest level among the phytochemicals assessed. Their presence in the extract suggests an important function in bioactivity and preservation potential, particularly in lipid‐rich systems where tannins could prevent oxidation.

**TABLE 5:** Major phytochemicals and antioxidant activity of prevailing synergistic extract (PSE).

Analyses	Prevailing synergistic extract (PSE)
Total flavonoids (mg QuE/mL)	1.27 ± 0.010
Total alkaloids (μg/g)	5.11 ± 0.067
Total tannins (mg TAE/g)	10.63 ± 0.513
DPPH IC_50_ (μg/mL)	23.04
ABTS IC_50_ (μg/mL)	30.71
FRAP (μg AAE/mg)	260.27 ± 2.050
TAC (μg AAE/mg)	345.20 ± 3.958

*Note:* Values are expressed as mean ± SD (*n* = 3), where applicable. IC_50_ values were calculated by nonlinear regression of concentration–response curves. No post hoc group comparison was applied because the table describes the optimized PSE only.

The antioxidant potential of the optimized PSE was thoroughly assessed through various in vitro assays, including DPPH and ABTS radical scavenging assays, alongside ferric reducing antioxidant power (FRAP) and total antioxidant capacity (TAC), as detailed in Table 5. The IC_50_ values for DPPH and ABTS radical scavenging activity were measured at 23.04 and 30.71 μg/mL, respectively, demonstrating a strong capacity for free radical quenching. The observed low IC_50_ values indicate the extract's effectiveness in neutralizing DPPH and ABTS radicals, with the DPPH results showcasing a more pronounced scavenging potential. The observed effect may be due to the synergistic interactions of polyphenolic compounds derived from the three medicinal plants, which improve the capacity for electron or hydrogen donation (Salimgareeva et al. 2024; Tsoupras et al. 2025).

The FRAP assay demonstrated a reducing power of 260.27 μg AAE/mg of extract, validating the extract's ability to convert Fe^3+^ to Fe^2+^ via its electron‐donating components. The significant reducing power aligns with earlier findings regarding the antioxidant properties of the specific plant components. Furthermore, TAC was measured at 345.20 μg AAE/mg, which further supports the robust antioxidant profile of the PSE. The elevated TAC value underscores the extract's ability to effectively neutralize various reactive oxygen species (ROS) and aiding in the maintenance of redox balance (Dutra et al. 2025; Kiss et al. 2025).

The PSE shows high antioxidant activity through multiple mechanisms, laying the groundwork for using the extract to build functional food products or nutraceuticals to reduce oxidative stress.

### 3.5 Antimicrobial Activity and Minimal Inhibitory Concentration (MIC)

The evaluation of the PSE antimicrobial activity was conducted against specific bacterial and fungal strains, with comparisons made to standard antibiotics (Table 6). The findings indicated that the sample displayed significant antibacterial activity, especially against *Staphylococcus aureus* (30 mm), showing an inhibition zone comparable to that of gentamycin (30 mm), which suggests a robust efficacy against this pathogen. The sample demonstrated notable inhibition against *Bacillus subtilis* (24 mm), *Klebsiella pneumoniae* (22 mm), and *Salmonella typhimurium* (20 mm). Although the inhibition zones were slightly lower than those recorded for Gentamycin, they still reflect a significant antibacterial potential. The sample demonstrated significant antifungal activity by effectively inhibiting *Candida albicans* with a zone of inhibition measuring 26 mm, which is marginally larger than the 25 mm observed with Fluconazole, indicating its robust efficacy against yeast‐like fungi. Nonetheless, the sample demonstrated no inhibitory effect against *Aspergillus niger* (Ghareeb et al. 2015; Helen et al. 2012), while fluconazole presented a distinct inhibition zone (25 mm), suggesting that the antifungal activity of the sample may be restricted to certain fungal strains.

**TABLE 6:** Antimicrobial activity and minimal inhibitory concentration (MIC) of prevailing synergistic extract (PSE).

Microorganism	Inhibition zone (mm)	MIC (μg/mL)
*Bacillus subtilis* (ATCC 6633)	24 ± 2^c^	31.25 ± 1^b^
*Staphylococcus aureus* (ATCC 6538)	30 ± 1^a^	15.62 ± 1^a^
*Klebsiella pneumoniae* (ATCC 13883)	22 ± 1^cd^	31.25 ± 1^b^
*Salmonella typhimurium* (ATCC 6539)	20 ± 2^d^	62.5 ± 1^c^
*Candida albicans* (ATCC 10221)	26 ± 1^b^	31.25 ± 2^b^
*Aspergillus niger* (ATCC 16888)	—	—

*Note:* Values are expressed as mean ± SD (*n* = 3). Different lowercase superscript letters within the same column indicate significant differences according to one‐way ANOVA followed by Tukey's HSD test at *p* < 0.05 (Table S5).

Abbreviation: MIC, minimal inhibitory concentration.

The determined minimal inhibitory concentration (MIC) values of the sample against chosen bacterial and fungal strains provide additional evidence of its strong antimicrobial activity (Table 6). The result recorded was 15.62 μg/mL for *Staphylococcus aureus* , demonstrating significant antibacterial effectiveness at low concentrations. Both *Bacillus subtilis* and *Klebsiella pneumoniae* demonstrated MIC values of 31.25 μg/mL, indicating similar susceptibility, even with their distinct Gram classifications. In a similar manner, *Candida albicans* exhibited a MIC of 31.25 μg/mL, further supporting the antifungal potential of the sample. *Salmonella typhimurium* demonstrated the highest MIC (62.5 μg/mL), suggesting a comparatively lower susceptibility in relation to the other microorganisms tested. This is likely attributed to its cell wall structure, which serves as a protective barrier against antimicrobial agents (Punchihewage‐Don et al. 2024). The findings are consistent with the inhibition zone data, indicating that the sample demonstrates notable efficacy against Gram‐positive bacteria and *Candida albicans* , although it necessitates higher concentrations to inhibit *Salmonella typhi* . The extensive antimicrobial capabilities of the sample underscore its potential as a natural antimicrobial agent.

### 3.6 Anti‐Inflammatory

**FIGURE 2:**
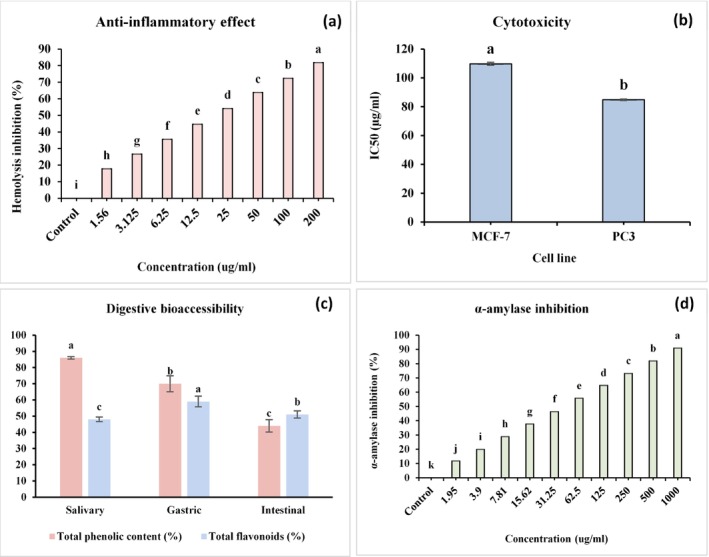
Bioactivity of prevailing synergistic extract (PSE): (a) Anti‐inflammatory effect; (b) cytotoxicity; (c) digestive bioaccessibility; (d) α‐Amylase inhibition. Data were analyzed by one‐way ANOVA followed by Tukey's HSD test for multiple‐group comparisons or Dunnett's post hoc test for comparisons against a single untreated/control group. Statistical significance was set at *p* < 0.05 (Table S5).

The findings from the bovine serum albumin (BSA) denaturation assay demonstrate a significant concentration‐dependent anti‐inflammatory effect of PSE (Figure 2a). The highest tested concentration of 200 μg/mL resulted in an inhibition of protein denaturation at 81.8%, while the lowest concentration of 1.56 μg/mL showed a reduction in inhibition to 17.8%. The observed gradual incline in inhibition as concentration increases clearly illustrates a dose–response relationship, indicating that higher concentrations offer enhanced protection against protein denaturation. The IC_50_ value was established at 18.21 μg/mL, indicating the sample's significant effectiveness as an inhibitor of BSA denaturation. A lower IC_50_ value indicates that a minimal concentration of the sample is sufficient to reach 50% inhibition, underscoring its efficacy in stabilizing protein structures and preventing denaturation, which is a crucial mechanism in inflammatory processes.

### 3.7 Cytotoxicity

The cytotoxic potential of PSE was assessed in vitro against MCF‐7 (breast) and PC3 (prostate) cancer cell lines (Figure 2b). The IC_50_ values recorded were 109.76 μg/mL for MCF‐7 cells and 84.82 μg/mL for PC3 cells, suggesting a moderate level of cytotoxic activity. The extract showed enhanced inhibitory effectiveness against PC3 cells, indicated by the reduced IC_50_ value, implying a greater sensitivity of prostate cancer cells to the bioactive compounds found in the PSE. This selective cytotoxicity could be linked to particular phytochemicals, including tanshinones derived from SMR and flavonoids from GBL, which have been documented to trigger apoptosis and suppress proliferation in prostate cancer models (Li et al. 2022).

The extract showed reduced efficacy against MCF‐7 cells (IC_50_ = 109.76 μg/mL), suggesting an antiproliferative effect linked to oxidative stress modulation or mitochondrial dysfunction, as seen in similar studies of phenolic‐rich botanical extracts.

Microscopic examination of treated MCF‐7 and PC3 cancer cells (Figures S3 and S4) demonstrated clear morphological alterations linked to cytotoxicity, thereby reinforcing the findings from the MTT assay. Significant changes in the structure of the cell surface and the cytoskeleton were noted, which were associated with decreased cell viability. Furthermore, distinct morphological features indicative of necrosis and apoptosis were observed. Cells undergoing necrosis displayed notable nuclear swelling, chromatin flocculation, and a reduction in nuclear basophilia, signifying significant and irreversible cellular damage. In contrast, cells undergoing apoptosis exhibited shrinkage, condensation of the nucleus, and fragmentation of nuclear material, indicating that the extract might trigger programmed cell death alongside necrosis. The results indicate the extract's cytotoxic potential and emphasize its capacity to activate various cell death pathways, warranting further investigation for targeted cancer treatment.

### 3.8 Digestive Bioaccessibility

The findings reveal notable differences in the bio‐accessibility of total phenolics and flavonoids extracted from PSE during the digestive process (Figure 2c). During the salivary phase, phenolic compounds demonstrated a high level of bio‐accessibility at 86%, whereas flavonoids showed a moderate availability of 48%. During the gastric phase of digestion, a significant reduction in phenolic content was observed, decreasing to 70%. This decline can likely be linked to the acidic environment's impact on phenolic stability. The decrease in phenolic bioaccessibility observed from the salivary to intestinal phase is in agreement with previous in vitro digestion studies showing that gastrointestinal conditions can substantially modify the stability and recovery of plant polyphenols. Rusak et al. (2021) reported marked digestion‐related changes in total phenolics, flavonoids, and antioxidant activity in green tea extracts, whereas Pinto et al. (2024) found that chestnut‐shell phenolics retained only about 30% bioaccessibility after digestion. In contrast, the bio‐accessibility of flavonoids rose to 59%, indicating a possible enhancement in their release or transformation in these conditions. During the intestinal phase, a notable reduction in phenolics to 44%, probably resulting from further degradation or complexation with other digestive elements, while flavonoid levels experienced a slight decrease to 51%. These findings demonstrate the dynamic nature of bioactive compounds release and change during digestion, showing that matrix interactions and gastrointestinal tract pH affect those compounds' accessibility.

### 3.9 In Vitro α‐Amylase Inhibition

The sample demonstrated a distinct concentration‐dependent effect on α‐amylase activity (Figure 2d), where increased concentrations of PSE led to enhanced enzyme inhibition. At the maximum concentration tested (1000 μg/mL), the inhibition of amylase was observed to be 90.9%, whereas at the minimal concentration (1.95 μg/mL), the inhibition decreased to 11.7%. The observed gradual incline in inhibition as sample concentration increases suggests a direct correlation between the availability of the inhibitor and the suppression of enzyme activity. The IC_50_ value was found to be 40.58 μg/mL, indicating that a comparatively low concentration of PSE is adequate to attain 50% inhibition of amylase activity. This inhibitory effect shows that the sample is an amylase inhibitor, which may regulate starch digestion and be used in functional food formulations or postprandial glucose management strategies.

The observed α‐amylase inhibitory activity provides an important in vitro biofunctional indication of the potential role of the optimized extract in modulating starch digestion. Inhibition of α‐amylase may slow the enzymatic hydrolysis of starch into smaller glucose‐releasing carbohydrates, thereby reducing the rate of glucose liberation during digestion and potentially contributing to a lower postprandial glycemic response. However, this finding should not be interpreted as direct evidence of an in vivo antidiabetic effect since the assay was performed under controlled enzymatic conditions and does not account for the complexity of food matrix interactions, gastrointestinal digestion, absorption, metabolism, or individual physiological responses. Recent fortified‐food research has emphasized that enzyme inhibition results become more nutritionally meaningful when they are integrated with actual food formulation and glycemic‐response evaluation. For example, Kafantari et al. (2025) linked the biofunctional properties of date fruit extracts with glycemic‐response outcomes after their incorporation into fortified muffins. Therefore, the present α‐amylase inhibition result suggests promising applicability of the optimized extract as a functional ingredient in starch‐rich foods, but further validation is required. Future studies should incorporate the extract into suitable cereal‐ or starch‐based model foods and evaluate in vitro starch digestibility, predicted glycemic index, texture, sensory acceptability, and phenolic stability after processing and storage to confirm its practical relevance for developing fortified foods aimed at glycemic‐response management.

## 4 Limitations

This study has several limitations that should be considered when interpreting the findings. First, the botanical materials were purchased commercially in freeze‐dried form, and the original lyophilization conditions were not available from the supplier; therefore, some variability in the initial phytochemical profile cannot be excluded. Second, all biological assays were conducted in vitro, including antioxidant, anti‐inflammatory, antimicrobial, cytotoxicity, α‐amylase inhibitory, and simulated gastrointestinal digestion tests, so the results should not be interpreted as direct evidence of in vivo efficacy. Third, the synergistic effect was assessed mainly through total phenolic content and selected bioactivities, without mechanistic interaction studies at the compound level. Fourth, although HPLC identified several phenolic constituents, other potentially relevant constituents of the herbal blend were not comprehensively profiled. Finally, the static INFOGEST digestion model cannot reproduce absorption, metabolism, gut microbiota transformation, or dynamic digestive conditions in humans. Accordingly, the conclusions have been moderated to emphasize in vitro potential rather than confirmed physiological effectiveness.

In addition, the RSM models were developed from a defined central composite design and are therefore valid primarily within the tested factor ranges. Although model fit was strong, external validation with additional batches and scale‐up trials is required before industrial application. Future validation should include independent botanical batches from different suppliers, standardized authentication, moisture content, particle‐size distribution, storage history, and targeted marker‐compound quantification before extraction optimization.

## 5 Future Perspectives

Future mechanistic studies should provide a deeper evaluation of the synergistic behavior of the optimized tri‐herbal extract. This should include testing each single‐plant extract, binary mixtures, the complete tri‐herbal mixture, chromatographic fractions, and recombined fractions at multiple concentration ratios. The nature of the interactions should then be quantified using appropriate models, such as Bliss independence, Loewe additivity, isobologram analysis, fractional inhibitory concentration index for antimicrobial effects, and the Chou–Talalay combination index for concentration–response assays. Such approaches would help clarify whether the observed effects are synergistic, additive, or antagonistic across different bioactivities and concentration ranges. Studies should also focus on translating the optimized extract into real functional food systems. This should include incorporation into food matrices, evaluation of processing and storage stability, sensory assessment, investigation of interactions with proteins, starch, lipids, and dietary fibers, and assessment of digestion behavior after formulation. The optimized extract should be tested in model food systems such as beverages, bakery products, dairy analogs, gels, or protein‐rich foods to determine its practical stability, functionality, and acceptability.

In addition, encapsulation by spray‐drying or freeze‐drying may improve phenolic stability, handling properties, and delivery efficiency. Complementary non‐thermal technologies, such as cold plasma processing, may also be explored to improve microbial safety and preserve functional quality, provided that possible phenolic degradation and sensory changes are carefully monitored. The present findings are also consistent with the broader valorization approach, in which phenolic‐rich plant extracts can be used as functional ingredients to improve antioxidant capacity, microbial stability, and shelf‐life of food systems. However, translation from extract‐level bioactivity to food application requires validation in real matrices, since pH, water activity, protein–phenolic interactions, lipid content, carbohydrates, minerals, and processing conditions may alter phenolic stability, bioaccessibility, color, flavor, and biological activity.

Finally, future development should include in vivo studies, toxicological assessment, standardization of marker compounds, stability testing, and jurisdiction‐specific regulatory evaluation before health‐related or commercial claims can be made.

## 6 Conclusion

In summary, the joint extraction of *Ginkgo biloba* leaves, *Astragalus membranaceus* roots, and *Salvia miltiorrhiza* roots demonstrated a synergistic effect, increasing phytochemical yield and enhancing bioactivity. The optimized tri‐herbal formulation showed greater total phenolic content and antioxidant capacity than expected from individual extracts, validating a genuine TPC‐based apparent synergistic interaction. The extract exhibited strong free‐radical scavenging, marked anti‐inflammatory effects in vitro, cytotoxic impacts on breast and prostate cancer cells, and potent antimicrobial properties against bacteria and yeast. Green extraction techniques, ultrasound and microwave, effectively concentrated bioactives while preserving their functionality, highlighting the advantages of sustainable methods. The extract's multifunctional properties support its use in functional foods and therapeutic phytochemical concentrates. In vitro bio‐accessibility confirmed phenolic availability post‐digestion, reinforcing in vivo potential.

## Author Contributions

**Mohamed Ibrahim Younis:** conceptualization, investigation, writing – original draft, writing – review and editing, validation, methodology, software, formal analysis, project administration, resources, supervision, data curation. **Khaled Fahmy Mahmoud:** data curation, supervision, resources, project administration, formal analysis, software, methodology, validation, writing – review and editing, writing – original draft, investigation, conceptualization. **Rawaa H. Tlay:** conceptualization, methodology, software, data curation, supervision, formal analysis, validation, investigation, writing – original draft, writing – review and editing, project administration, resources. **Tarek Gamal Abedelmaksoud:** resources, supervision, data curation, software, formal analysis, project administration, writing – review and editing, validation, methodology, conceptualization, investigation, writing – original draft. **Yahia Ibrahim Sallam:** conceptualization, investigation, writing – original draft, writing – review and editing, validation, methodology, software, formal analysis, project administration, resources, supervision, data curation. **M. Ali Aboudzadeh:** conceptualization, investigation, writing – original draft, writing – review and editing, validation, methodology, software, formal analysis, project administration, resources, supervision, data curation.

## Conflicts of Interest

The authors declare no conflicts of interest.

## Supporting information


**Figure S1:** HPLC chromatogram of standard.
**Figure S2:** HPLC chromatogram of sample.
**Figure S3:** Effect of PSE on MCF‐7 cells at different concentrations.
**Figure S4:** Effect of PSE on PC3 cells at different concentrations.
**Table S1:** Extraction conditions affecting total phenolic content yield (mg GAE/g).
**Table S2:** Experimental design matrix of the central composite design (CCD).
**Table S3:** Statistical evaluation of RSM models for phenolic extraction using green techniques.
**Table S4:** Lack‐of‐fit test results from ANOVA for RSM models.
**Table S5:** One‐way ANOVA results for all measured parameters.

## Data Availability

Data will be made available on request.
