# Supplementary material for: From Green Extraction to Gut Bioaccessibility: Synergistic Potential of Ginkgo biloba, Astragalus membranaceus, and Salvia miltiorrhiza Phytochemicals for Functional Food Applications
Source: Food Sci Nutr. 2026 Jun 29;14(7):e72045. doi: 10.1002/fsn3.72045 (PMC13312308; doi:10.1002/fsn3.72045)
Supplement: Supplementary file 1 — Figure S1: HPLC chromatogram of standard. Figure S2: HPLC chromatogram of sample. Figure S3: Effect of PSE on MCF‐7 cells at different concentrations. Figure S4: Effect of PSE on PC3 cells at different concentrations. Table S1: Extraction conditions affecting total phenolic content yield (mg GAE/g). Table S2: Experimental design matrix of the central composite design (CCD). Table S3: Statistical evaluation of RSM models for phenolic extraction using green techniques. Table S4: Lack‐of‐fit test results from ANOVA for RSM models. Table S5: One‐way ANOVA results for all measured parameters. [file FSN3-14-e72045-s001.docx]

**From Green Extraction to Gut Bioaccessibility: Synergistic Potential of *Ginkgo biloba*, *Astragalus membranaceus*, and *Salvia miltiorrhiza* Phytochemicals for Functional Food Applications**

Mohamed Ibrahim Younis^1^, Yahia Ibrahim Sallam^1^, Khaled Fahmy Mahmoud^2^, Rawaa H. Tlay^3*^, M. Ali Aboudzadeh^4^, Tarek Gamal Abedelmaksoud^1*^

**Supplementary description**

**Figures:**


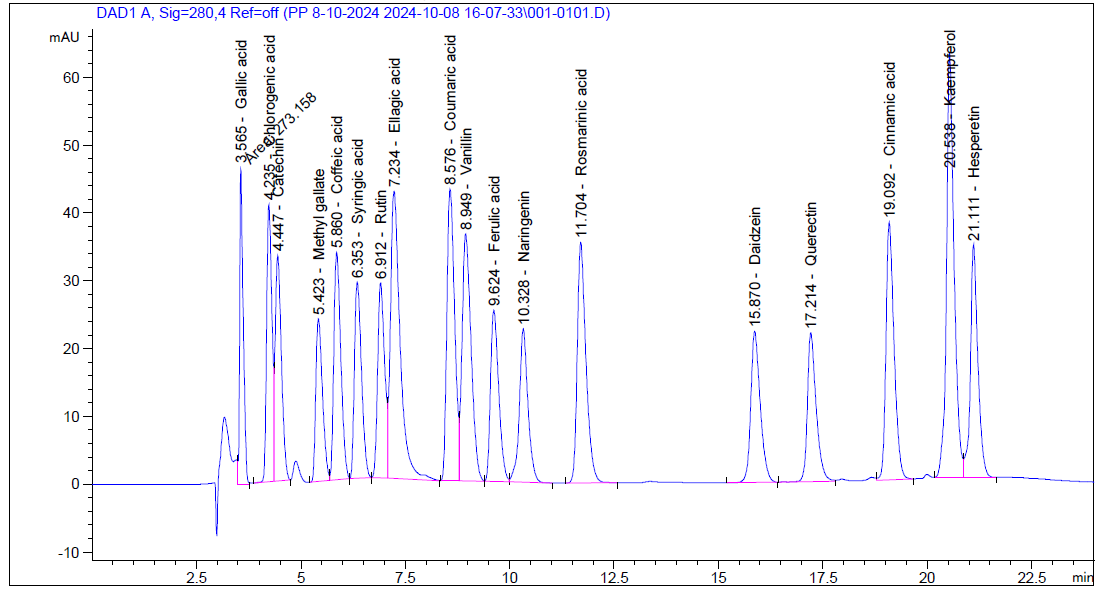


**Figure S1.** HPLC chromatogram of standard


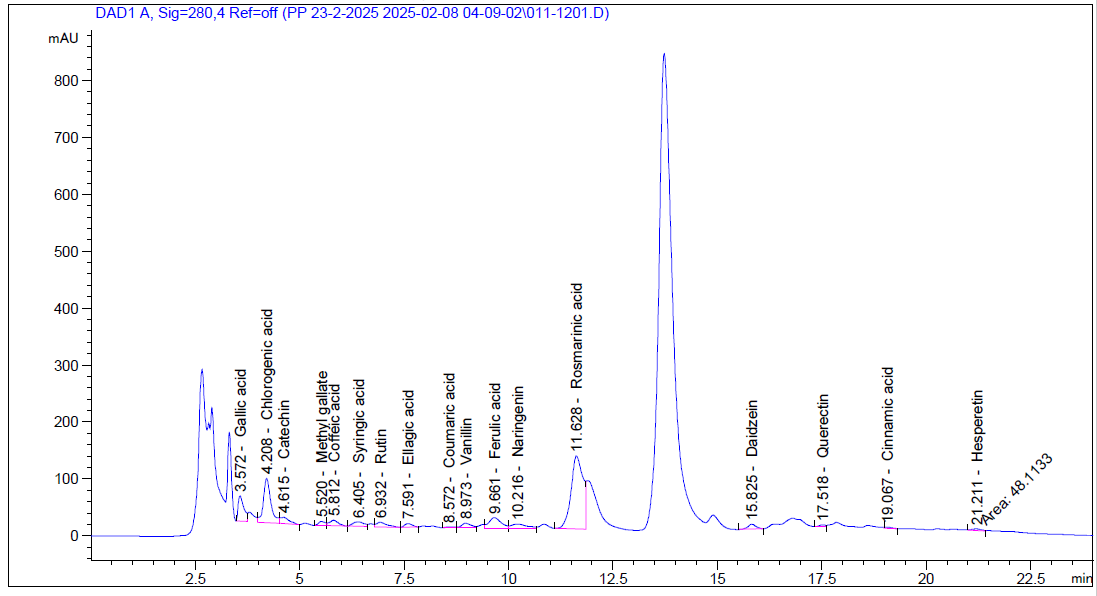


**Figure S2.** HPLC chromatogram of sample


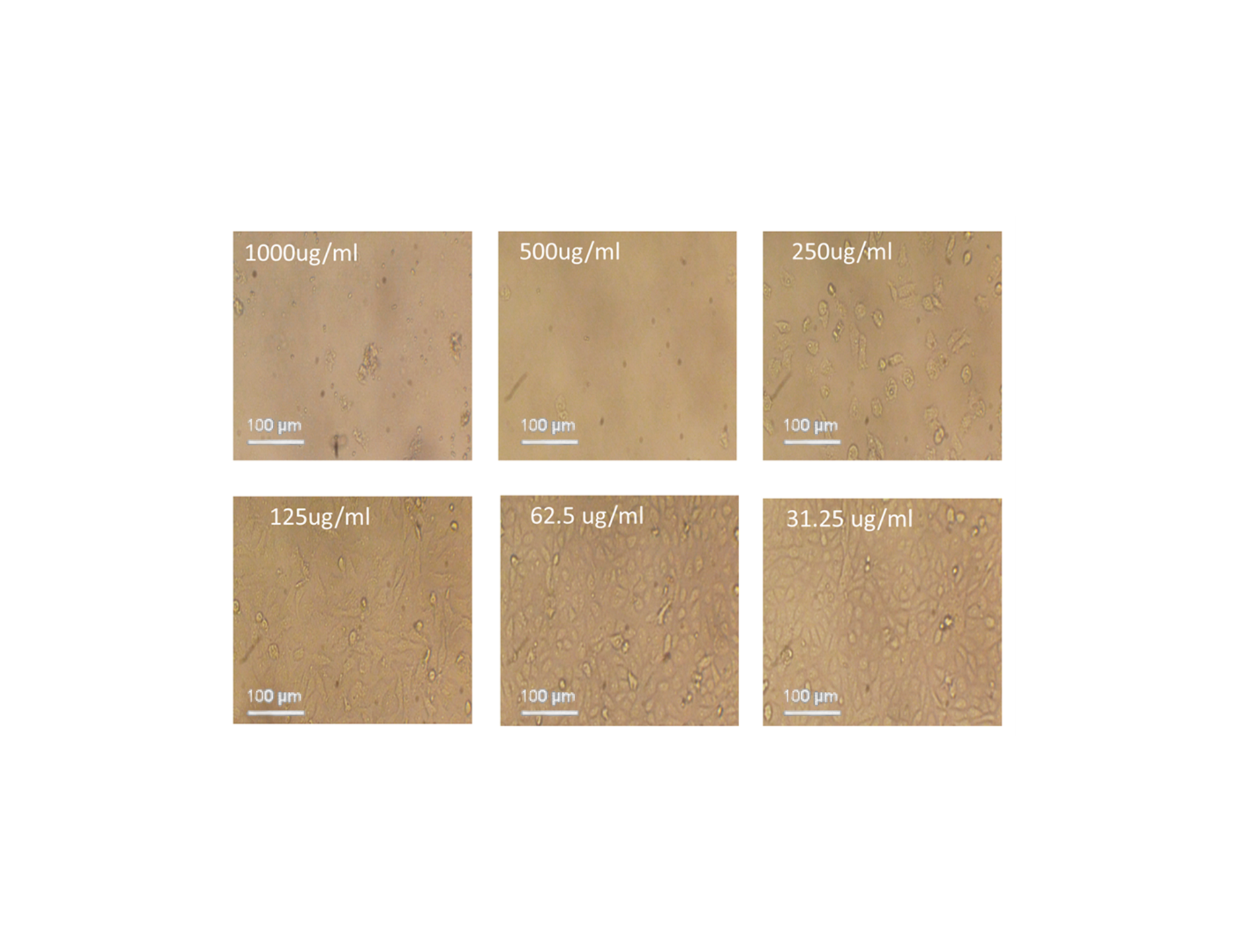


**Figure S3.** Effect of PSE on MCF-7 cells at different concentrations


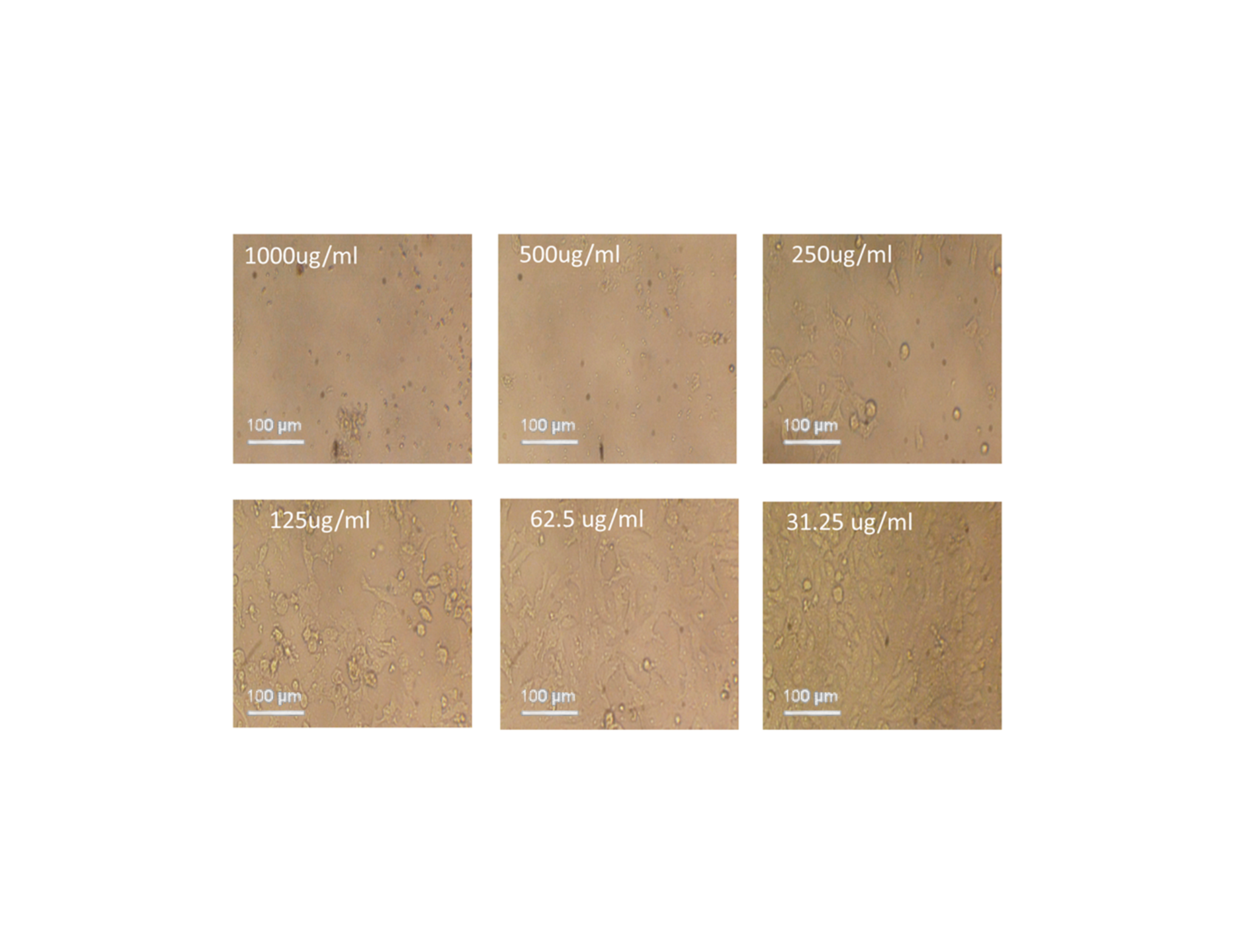


**Figure S4.** Effect of PSE on PC3 cells at different concentrations

**Tables:**

**Table S1.** Extraction conditions affecting total phenolic content yield (mg GAE/g).

| **Extraction method** | **Conditions** | |
| --- | --- | --- |
|  | **X_1_** | **X_2_** |
| **Probe ultrasonication** | Sonication power (W) | Time (min) |
| **Bath ultrasonication** | Time (min) | Temperature (⁰C) |
| **Microwave-assisted** | Time (min) | Microwave power (W) |

**Table S2.** Experimental design matrix of the central composite design (CCD).

| **Run** | **Probe ultrasonication** | | **Bath ultrasonication** | | **Microwave-assisted** | |
| --- | --- | --- | --- | --- | --- | --- |
|  | **X_1_** | **X_2_** | **X_1_** | **X_2_** | **X_1_** | **X_2_** |
| **1** | 100 | 10 | 10 | 30 | 5 | 200 |
| **2** | 400 | 10 | 40 | 30 | 5 | 800 |
| **3** | 250 | 10 | 10 | 60 | 30 | 200 |
| **4** | 250 | 60 | 40 | 60 | 30 | 800 |
| **5** | 400 | 35 | 10 | 45 | 2.5 | 500 |
| **6** | 100 | 60 | 40 | 45 | 32.5 | 500 |
| **7** | 250 | 60 | 25 | 30 | 17.5 | 100 |
| **8** | 250 | 35 | 25 | 60 | 17.5 | 900 |
| **9** | 340 | 35 | 25 | 45 | 17.5 | 500 |
| **10** | 100 | 35 | 25 | 45 | 17.5 | 500 |
| **11** | 250 | 35 | 25 | 45 | 17.5 | 500 |
| **12** | 340 | 10 | 5 | 45 | 17.5 | 200 |
| **13** | 340 | 50 | 45 | 45 | 17.5 | 500 |

**Table S3.** Statistical evaluation of RSM models for phenolic extraction using green techniques.

| **Parameter** | **Extraction method** | | |
| --- | --- | --- | --- |
|  | **Probe ultrasonication** | **Bath ultrasonication** | **Microwave-assisted** |
| **Std. Dev** | 0.3526 | 2.13 | 0.8456 |
| **Mean** | 332.28 | 264.88 | 192.21 |
| **C.V.%** | 0.1061 | 0.8023 | 0.44 |
| **PRESS** | 3.05 | 158.36 | 14.59 |
| **R^2^** | 0.9991 | 0.9808 | 0.9990 |
| **Adj R^2^** | 0.9985 | 0.9671 | 0.9983 |
| **Pred R^2^** | 0.9969 | 0.9039 | 0.9971 |
| **Adeq Precision** | 127.6556 | 28.7913 | 90.3470 |

**Table S4.** Lack-of-fit test results from ANOVA for RSM models.

| **Lack of fit values** | **Extraction method** | | |
| --- | --- | --- | --- |
|  | **Probe ultrasonication** | **Bath ultrasonication** | **Microwave-assisted** |
| **Sum of squares** | 0.8702 | 28.06 | 4.13 |
| **Degree of freedom** | 5 | 5 | 4 |
| **Mean square** | 0.1740 | 5.61 | 1.03 |
| **F-value** | - | 3.16 | 3.52 |
| **p-vlaue** | - | 0.2576 | 0.1646 |

Lack-of-fit F- and p-values for probe ultrasonication were not estimated due to zero pure error resulting from identical replicated responses.

**Table S5.** One-way ANOVA results for all measured parameters.

| **Parameter** | **F-value** | **df (between, within)** | **p-value** | **Significance** |
| --- | --- | --- | --- | --- |
| **Fat** | 50.3496 | (2, 6) | 1.78 × 10⁻⁴ | *** |
| **Ash** | 147.5129 | (2, 6) | 7.92 × 10⁻⁶ | *** |
| **Total Carbohydrates** | 32.4345 | (2, 6) | 6.07 × 10⁻⁴ | *** |
| **Protein** | 602.8929 | (2, 6) | 1.21 × 10⁻⁷ | *** |
| **Inhibition zone** | 20.1818 | (4, 10) | 8.86 × 10⁻⁵ | *** |
| **MIC** | 549.4043 | (4, 10) | 1.14 × 10⁻¹¹ | *** |
| **Anti-inflammatory** | 1.99 × 10⁸ | (8, 18) | 6.59 × 10⁻⁷⁰ | *** |
| **Cytotoxicity** | 1318.4560 | (1, 4) | 3.43 × 10⁻⁶ | *** |
| **Bioaccessability (TPC)** | 96.2857 | (2, 6) | 2.76 × 10⁻⁵ | *** |
| **Bioaccessability (TF)** | 15.7297 | (2, 6) | 0.00411 | ** |
| **α-amylase inhibition** | 2.49 × 10⁸ | (10, 22) | 3.50 × 10⁻⁸⁶ | *** |

MIC: Minimal inhibitory concentration; TPC: Total phenolic content; TF: Total flavonoids; n = 3 per group; df : degrees of freedom; Significance levels: *** p < 0.001, ** p < 0.01, and * p < 0.05.
